# Performance of clinical signs and symptoms, rapid and reference laboratory diagnostic tests for diagnosis of human African trypanosomiasis by passive screening in Guinea: a prospective diagnostic accuracy study

**DOI:** 10.1186/s40249-023-01076-1

**Published:** 2023-03-20

**Authors:** Oumou Camara, Mamadou Camara, Laura Cristina Falzon, Hamidou Ilboudo, Jacques Kaboré, Charlie Franck Alfred Compaoré, Eric Maurice Fèvre, Philippe Büscher, Bruno Bucheton, Veerle Lejon

**Affiliations:** 1 Programme National de Lutte contre la Trypanosomiase Humaine Africaine, Conakry, Guinea; 2 International Livestock Research Institute, Nairobi, Kenya; 3 Institute of Infection, Veterinary and Ecological Sciences, University of Liverpool, Liverpool, UK; 4 Clinical Research Unit of Nanoro, Institute for Health Science Research (IRSS), Ouagadougou, Burkina Faso; 5 Vector-Borne Diseases and Biodiversity Unit, International Research and Development Center on Livestock in Sub-Humid Areas (CIRDES), Bobo-Dioulasso, Burkina Faso; 6 Unit of Research and Training in Life and Earth Sciences, University of Nazi Boni, Bobo-Dioulasso, Burkina Faso; 7 Department of Biomedical Sciences, Institute of Tropical Medicine, Antwerp, Belgium; 8 UMR Intertryp IRD-CIRAD, French National Research Institute for Sustainable Development (IRD), University of Montpellier, Montpellier, France

**Keywords:** Human African trypanosomiasis, *Trypanosoma brucei gambiense*, Diagnosis, Clinical, Rapid diagnostic test, Sensitivity, Specificity, Dried blood spot, Trypanolysis

## Abstract

**Background:**

Passive diagnosis of human African trypanosomiasis (HAT) at the health facility level is a major component of HAT control in Guinea. We examined which clinical signs and symptoms are associated with HAT, and assessed the performance of selected clinical presentations, of rapid diagnostic tests (RDT), and of reference laboratory tests on dried blood spots (DBS) for diagnosing HAT in Guinea.

**Method:**

The study took place in 14 health facilities in Guinea, where 2345 clinical suspects were tested with RDTs (HAT Sero-*K*-Set, rHAT Sero-Strip, and SD Bioline HAT). Seropositives underwent parasitological examination (reference test) to confirm HAT and their DBS were tested in indirect enzyme-linked immunoassay (ELISA)/*Trypanosoma brucei gambiense*, trypanolysis, Loopamp *Trypanosoma brucei* Detection kit (LAMP) and m18S quantitative PCR (qPCR). Multivariable regression analysis assessed association of clinical presentation with HAT. Sensitivity, specificity, positive and negative predictive values of key clinical presentations, of the RDTs and of the DBS tests for HAT diagnosis were determined.

**Results:**

The HAT prevalence, as confirmed parasitologically, was 2.0% (48/2345, 95% *CI:* 1.5–2.7%). Odds ratios (*OR*) for HAT were increased for participants with swollen lymph nodes (*OR* = 96.7, 95% *CI:* 20.7–452.0), important weight loss (*OR* = 20.4, 95% *CI*: 7.05–58.9), severe itching (*OR* = 45.9, 95% *CI*: 7.3–288.7) or motor disorders (*OR* = 4.5, 95% *CI*: 0.89–22.5). Presence of at least one of these clinical presentations was 75.6% (95%* CI*: 73.8–77.4%) specific and 97.9% (95% *CI*: 88.9–99.9%) sensitive for HAT. HAT Sero-*K*-Set, rHAT Sero-Strip, and SD Bioline HAT were respectively 97.5% (95% *CI*: 96.8–98.1%), 99.4% (95% *CI*: 99.0–99.7%) and 97.9% (95% *CI*: 97.2–98.4%) specific, and 100% (95% *CI*: 92.5–100.0%), 59.6% (95% *CI*: 44.3–73.3%) and 93.8% (95% *CI*: 82.8–98.7%) sensitive for HAT. The RDT’s positive and negative predictive values ranged from 45.2–66.7% and 99.2–100% respectively. All DBS tests had specificities ≥ 92.9%. While LAMP and m18S qPCR sensitivities were below 50%, trypanolysis and ELISA/*T.b. gambiense* had sensitivities of 85.3% (95%* CI*: 68.9–95.0%) and 67.6% (95% *CI*: 49.5–82.6%).

**Conclusions:**

Presence of swollen lymph nodes, important weight loss, severe itching or motor disorders are simple but accurate clinical criteria for HAT referral in HAT endemic areas in Guinea. Diagnostic performances of HAT Sero-*K*-Set and SD Bioline HAT are sufficient for referring positives to microscopy. Trypanolysis on DBS may discriminate HAT patients from false RDT positives.

*Trial registration* The trial was registered under NCT03356665 in clinicaltrials.gov (November 29, 2017, retrospectively registered https://clinicaltrials.gov/ct2/show/NCT03356665)

**Graphical Abstract:**

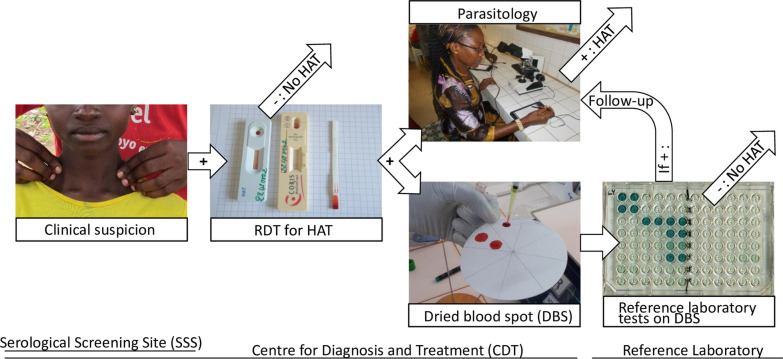

## Background

Infection with the parasite *Trypanosoma brucei gambiense* (*T.b. gambiense*) causes the chronic form of human African trypanosomiasis (HAT), also called sleeping sickness. While in Central Africa, the Democratic Republic of the Congo is responsible for about three quarters of all reported gambiense HAT patients, in West Africa, Guinea is frontrunner in the number of cases [1]. Almost all Guinean HAT cases occur along the coastline, in particular in the prefectures of Boffa, Dubreka and Forecariah [2, 3].

Despite considerable challenges, Guinea implements an efficacious HAT control program based on medical interventions supplemented with vector control. Even during the Ebola epidemic outbreak in 2014–2016, the national HAT control program managed to deploy insecticide impregnated targets in Boffa, and limited parasite transmission to humans by reducing the tsetse fly vector density [4]. Medical interventions against HAT in Guinea consist of passive and active screening, followed by treatment of confirmed HAT cases. During active screening, a specialized team visits the most affected villages and tests the whole population. An important drawback, however, is that once the disease prevalence drops, cost-effectiveness of active screening decreases as fewer cases are detected [5]. Also, as experienced in Guinea in the recent past, during epidemics of other infections, active screening may be interrupted [6, 7]. While approaching the status of elimination of HAT as a public health problem, passive screening for HAT, integrated in the existing health system, therefore increases in importance, is more resilient to interruption and more sustainable. In Guinea, passive screening was maintained at a low level during the Ebola epidemic, and was rapidly resumed in 2016 [3]. In passive screening, serological testing for HAT among individuals consulting a health centre, is initiated by the observation of symptoms or signs considered “suggestive” for HAT [3]. In such clinical suspects, an antibody detection test, usually a rapid diagnostic test (RDT), is carried out. Subjects that are RDT positive are subsequently examined microscopically for trypanosome presence, while those that test RDT negative, are considered HAT free. As neither the RDT positive predictive values (PPV) nor the sensitivities of parasite detection techniques are 100%, not all RDT positive suspects are parasitologically confirmed. To further discriminate potential HAT patients from RDT false positives and better target additional labour-intensive microscopic re-examinations, further reference laboratory tests can be carried out remotely on dried blood spots (DBS).

While the most sensitive parasite detection techniques are routinely applied in Guinea [3, 8], for nearly all the other steps of the diagnostic chain, different options to increase or decrease suspicion for HAT are available, which, depending on their HAT diagnostic performance, influence effectiveness of screening. Although the clinical picture of gambiense HAT is relatively well documented [9, 10], the association of signs and symptoms with HAT in a healthcare seeking population has hardly been studied [11]. Furthermore, in the last decade, several RDTs have emerged for individual screening of HAT clinical suspects [12, 13]. For DBS testing, trypanolysis and enzyme-linked immunoassay (ELISA)/*T.b. gambiense* are available to detect antibodies against *T.b. gambiense* [14–16], while *Trypanozoon* specific DNA can be detected using the Loopamp *Trypanosoma brucei* Detection kit (LAMP) or m18S quantitative PCR (qPCR) [17, 18]. So far, for Guinea, the diagnostic performance of SD Bioline HAT, HAT Sero-*K*-Set and trypanolysis has mainly been evaluated retrospectively on stored plasma specimens or DBS [14, 19].

Within the framework of a multi-country diagnostic trial, the diagnostic performance of clinical signs and symptoms, of 3 RDTs and of serological and molecular reference laboratory tests on DBS was evaluated prospectively for diagnosis of HAT, in the context of passive screening in HAT endemic areas in Guinea.

## Methods

### Study setting

In Guinea, clinical suspects were prospectively recruited for the Diagnostic tools for human African trypanosomiasis elimination and clinical trials work package 2 (DiTECT-HAT-WP2) study between January 2017 and January 2020 in 14 hospitals and health posts in the prefectures of Boffa, Dubreka and Forecariah. In these 3 prefectures, the HAT prevalence expressed as number of HAT cases per 10,000 inhabitants in 2017 was respectively 2.92, 0.53 and 1.51, and decreased to 0.97, 0.33 and 0.99 in 2019 [3]. Serological screening sites (SSS) offered clinical and serological screening for HAT, and referred participants who were RDT positive to a centre for diagnosis and treatment (CDT). The CDT performed parasitological examination of RDT positives, in addition to clinical and serological screening. In Boffa prefecture, participants were recruited in Boffa hospital that acted as the CDT, while the health posts of Soumbouyadi, Tamita and Walia acted as SSS. In Dubreka prefecture, Dubreka LTO was the CDT, with 3 SSS, the health centres of Dubreka CU, Magnokhoun and Kholoya. In Forecariah prefecture, the health centre of Karakoro acted as CDT, while the health centers of Benty, Konta, Madinagbe, M’Boro and Sinkinet were SSS. The number of people looking for medical consultation in these 14 structures was around 40,000 in total in 2020 (ranging between 430 and 25,306 for individual centres).

The initial sample size estimation was based on 13 health structures, performing RDTs on 15 clinical suspects each month, for 27 months, which would have resulted in 5265 inclusions. We estimated that in about 10% of the clinical suspects tested, at least one RDT would be positive. The HAT prevalence among clinical suspects was estimated at 1%, which would result in inclusion of 53 HAT patients.

### Study protocol

**Fig. 1:**
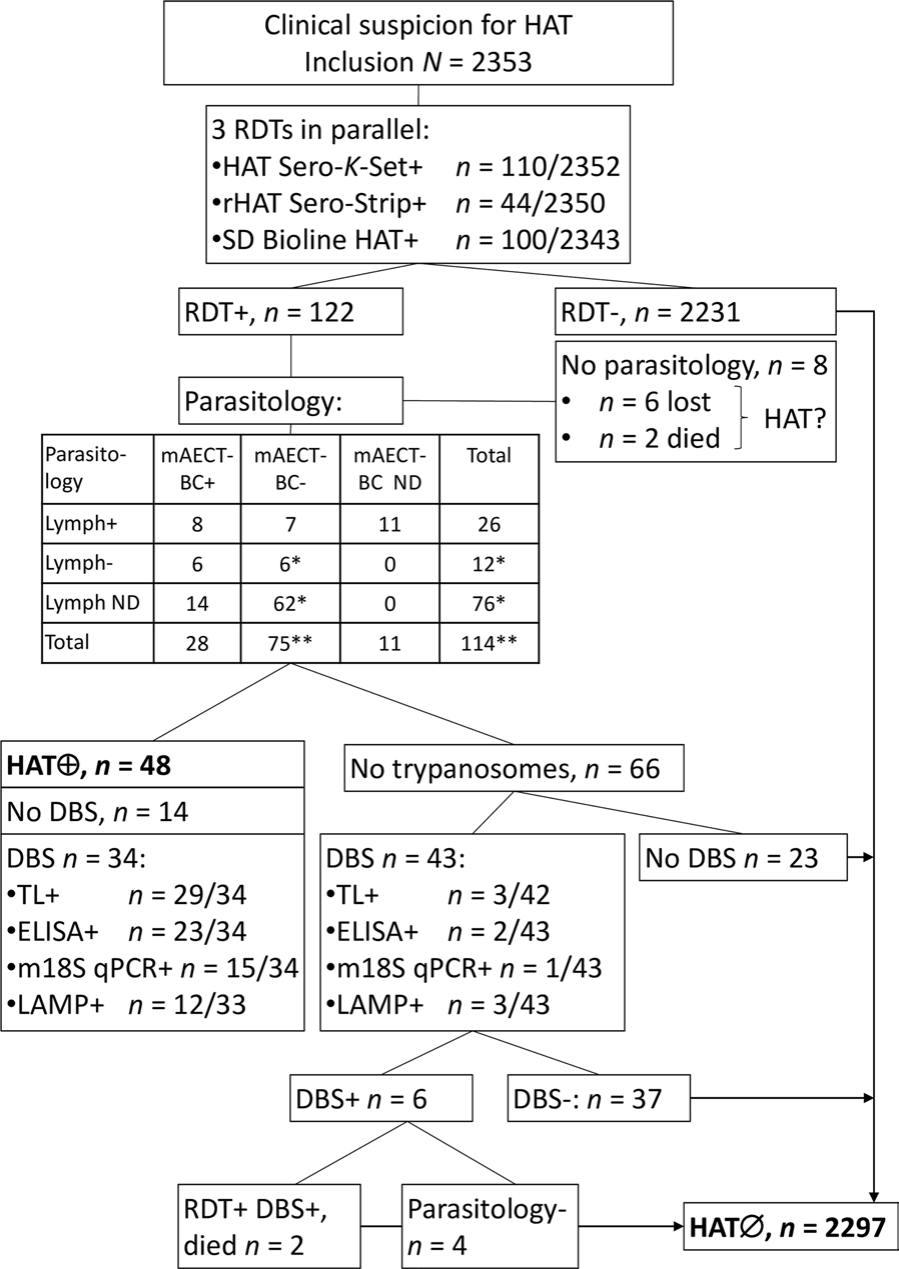
DiTECT-HAT-WP2 study conduct and test results in Guinea. *CSF* Cerebrospinal fluid; *DBS* dried blood spot; *HAT* Human African trypanosomiasis; *mAECT-BC* mini anion exchange centrifugation on buffy coat; *RDT* rapid diagnostic test. *HAT confirmed by CSF examination. ND: not done. HAT ⊕ : HAT positive. HAT∅: HAT free

The study protocol is summarized in Fig. 1.

#### Inclusion criteria

Individuals consulting the study SSS or CDT could be consecutively included if they had visited or resided in a HAT endemic area and presented with clinical suspicion for HAT. Clinical suspicion was defined as presence of at least one of the following clinical signs or symptoms: Recurrent fever not responding to anti-malarial medication; headache for a long duration (> 14 days); presence of enlarged lymph nodes in the neck; important weight loss; weakness; severe itching; amenorrhea, abortion, or sterility; coma; psychiatric problems (e.g., aggressiveness, apathy, mental confusion, increasing unusual hilarity, etc.); sleep disruption (nocturnal insomnia and/or excessive diurnal sleeping); motor disorders (abnormal movements, shaking, walking difficulties); convulsions; or speech disorders. Individuals were excluded from participation if they had already been treated for HAT, did not give their written informed consent or were less than 4 years old.

#### Serological screening and parasitological confirmation

Finger prick blood was tested with 3 RDTs, HAT Sero-*K*-Set (Coris Bioconcept, Gembloux, Belgium), rHAT Sero-Strip (Coris Bioconcept, Gembloux, Belgium) and SD Bioline HAT (Abbott, Yongin-si, Gyeonggi-do, the Republic of Korea) according to the instructions of the manufacturers. Clinical suspects negative in all 3 RDTs were considered HAT free, while those that were positive in at least one RDT, were considered serological suspects. Parasitological examination of serological suspects was carried out in the CDTs. If enlarged lymph nodes in the neck were present in the serological suspect, a lymph node puncture was performed, and a drop of lymph was microscopically examined under 10 × 40 magnification for presence of trypanosomes. From every serological suspect, venous blood on heparin was taken. If no lymphadenopathy was present or no parasites had been observed in lymph, 4 ml of heparinized blood was centrifuged, the buffy coat was taken and analysed for presence of trypanosomes using the mini anion exchange centrifugation technique on buffy coat (mAECT-BC) [8].

#### Dried blood spot preparation

For every serological suspect two types of DBS were prepared. On a Whatman grade 4 filter paper, 16 drops of 30 µl of heparinized blood were deposited and left to dry. In parallel, 180 µl of heparinized blood were lysed for 5 min with 20 µl of 5% SDS solution (Sigma Aldrich, Saint Louis, MO, USA), and 2 drops of 40 µl of lysed blood were deposited on a Whatman grade 1001 filter paper. Filter papers were dried, packed in separate envelopes, which in turn were packed in hermetic plastic bags containing silica gel.

#### Patient management

A lumbar puncture was carried out on parasitologically confirmed HAT patients, or if the clinician considered it appropriate, based on strong clinical suspicion. The cerebrospinal fluid (CSF) was examined for the number of white blood cells, and for presence of trypanosomes using the modified single centrifugation [20]. Patients with parasitologically confirmed HAT without CSF trypanosomes and white blood cell numbers ≤ 5/µl, were considered in first stage and HAT patients with > 5 white blood cells/µl or trypanosomes in CSF were classified as second stage. Treatment of HAT was carried out according to the treatment protocols in place at the CDTs.

#### Study participants with missing data

Serological suspects that could not be confirmed at the first microscopic examination, were invited for re-examination at the CDT or were re-examined by the national program. A number of RDT seropositives detected at SSS level and who did not show up at CDT were offered microscopic examination through the national sleeping sickness program (PNLTHA).

### Reference laboratory tests

The DBS were sent to the Centre International de Recherche-Développement sur l’Elevage en zone Subhumide (Bobo-Dioulasso, Burkina Faso), where reference laboratory tests were performed. Test performers were not informed about the clinical and reference standard results. On DBS collected on Whatman grade 4 paper, trypanolysis and ELISA/*T.b. gambiense* were carried out for *T.b. gambiense* specific antibody detection, both targeting LiTat 1.3 and 1.5 VSG, following the methodology previously described [21]. For *Trypanozoon* DNA detection, m18S qPCR was carried out on DBS collected on Whatman grade 4 and if positive, followed by TgsGp-qPCR, while the lysed blood collected on Whatman grade 1001 was tested with Loopamp *Trypanosoma brucei* Detection Kit (LAMP, Eiken Chemical, Tokyo, Japan), according to published methodologies [18].

### Data analysis

Results obtained at the CDT were immediately entered in a digital case report form [22]. The application incorporated demographic, clinical and diagnostic data, and included pictures of positive RDTs and videos of trypanosome positive microscopy results. Results from SSS were collected on a paper case report form and entered retrospectively in the application. Data were transferred from the application to a central database and exported into a Excel spreadsheet (Microsoft Office Professionnel Plus 2016). Descriptive statistics were carried out to check for missing data and variation in each variable; categorical variables were summarized as proportions, while continuous variables were summarized with the median value and range.

Regression analysis and evaluation of the diagnostic performance were based on the participants HAT status (Fig. 1). Participants with trypanosomes detected in lymph, blood or CSF were considered HAT positive. Participants who were triple RDT negative were considered HAT negative. Participants who were RDT positive but in whom no trypanosomes could be detected after microscopic examination(s), were considered HAT negative. Participants who were RDT positive, but did not undergo any parasitological examination were disregarded.

Regression analysis using Stata Statistical Software (Release 14, College Station, TX: StataCorp LP) was performed to assess for associations with the HAT status and thus identify which demographic features and clinical presentations could be used as criteria to target future testing for HAT. Continuous variables were assessed for normal distribution, and the correlation between the thirteen clinical signs and symptoms was determined. Unconditional associations between HAT status and the explanatory variables (gender, age, and clinical signs and symptoms) were investigated. Subsequently, mixed logistic regression models were developed for the HAT status, with prefecture included as a random effect to account for spatial clustering within each prefecture. Backward elimination was then used to screen variables, and only statistically significant variables (*P* ≤ 0.05) were retained. Two-way interaction terms between all remaining variables were assessed for statistical significance. The final multivariable model included variables that were either statistically significant, or were part of a significant interaction term [23]. The intra-cluster coefficient was computed as the proportion of overall variation due to variation between groups, while interaction terms were interpreted using the coefficients [24]. As an example, the odds of a patient being HAT positive when having both enlarged lymph nodes in the neck and severe itching (compared to a patient who had neither enlarged lymph nodes in the neck nor severe itching), was determined as follows: exp [Coefficient Enlarged lymph nodes in the neck + Coefficient severe itching + Coefficient Enlarged lymph nodes in the neck X Severe itching].

The diagnostic performance of the clinical presentation (only those that were retained in mixed logistic regression), the three RDTs (individually, in parallel, and in series), and of the four reference laboratory tests was determined. The sensitivity, specificity, positive predictive value (PPV), negative predictive value (NPV) and accuracy for diagnosis of HAT were calculated with 95% Clopper Pearson confidence intervals (GraphPad Prism 9). The Kappa agreement for combinations of RDTs and reference laboratory tests was also determined, and interpreted as poor (< 0.00), slight (0.00–0.20), fair (0.21–0.40), moderate (0.41–0.60), substantial (0.61–0.80), or almost perfect (0.81–1.00) [25].

## Results

### Descriptive statistics of field results

In total, 2353 clinical suspects were included: 707 in the prefecture of Boffa, 705 in Dubreka, and 941 in Forecariah. Of these clinical suspects, 1320 (56.1%) were female and 1033 (43.9%) male. Their median age was 30 years (range: 4–89). The most frequently observed clinical presentations were recurrent fever not responding to anti-malarial medication (96%) and headache for a long duration (80.3%), followed by weakness (21.1%) (Table 1). Overall, among the 2353 study participants (Fig. 1), 122 tested positive to at least one RDT (5.2%; 95% *CI*: 4.3–6.2%). Specifically, 110/2352 (4.7%; 95% *CI*: 3.9–5.6%) were positive to HAT Sero-*K*-Set; 44/2350 (1.9%, 95%* CI*: 1.4–2.5%) were positive to rHAT Sero-Strip; and 100/2343 (4.3%, 95% *CI*: 3.5–5.2%) were positive to SD Bioline HAT. Among the 122 RDT positives, 114 serological suspects were parasitologically examined (6 were lost to follow-up and 2 died before parasitology could be carried out). Forty-eight individuals were diagnosed with parasitologically confirmed HAT (48/2345, 2.0%; 95% *CI*: 1.5–2.7%), among whom 26 were trypanosome positive in lymph (26/48, 54.2%; 95% *CI*: 39.2–68.6%), and 28 in mAECT-BC (28/48, 58.3%; 95% *CI*: 43.2–72.4%). Out of 21 RDT positives for whom both lymph and blood were examined, eight tested positive in both body fluids. In MSC, 18/40 HAT patients tested had trypanosomes in CSF (45.0%; 95% *CI*: 29.3–61.5%), of which 2 had not been previously confirmed in lymph or blood (2/48, 4.2%; 95%* CI*: 0.5–14.3%). Three clinical suspects with positive RDTs were not parasitologically confirmed in blood or lymph and underwent lumbar puncture based on clinical suspicion, which enabled to confirm parasite presence by MSC in two. The remaining suspect (with recurrent fever not responding to anti-malarial medication, headache for a long duration, enlarged lymph nodes in the neck and motor disorders) had a white blood cell count of 265/µl, but in the absence of parasites, was not considered a HAT patient. The median CSF white blood cell count for HAT patients was 145/µl (range: 12–1086/µl). All HAT patients for whom CSF data were available (46/48) were in 2^nd^ stage. The HAT patients included 19 females (39.6%) and 29 males (60.4%), and their median age was 26 years (range: 10–65).

**Table 1:** Frequency of clinical presentations in study participants and HAT patients, and association to HAT positivity

Variable	All participants *n* = 2353	HAT *n* = 48	Univariable analysis		Multivariable mixed logistic regression	
	Frequency (number)	Frequency (number)	*P* value	OR (95% *CI*)	*P* value	OR (95% *CI*)
Male	43.9% (1033)	60.4% (29)	0.02	2.0 (1.1–3.5)	0.016	3.1 (1.2–7.6)
Age	NA	NA	0.02	0.98 (0.96–1.00)		
Recurrent fever not responding to anti-malarial medication	96.0% (2258)	89.6% (43)	0.03	0.4 (0.1–0.9)		
Headache for a long duration	80.3% (1889)	91.7% (44)	0.06	2.7 (0.07–7.7)		
Weakness	21.1% (496)	54.2% (26)	< 0.001	4.6 (2.6–8.2)		
Important weight loss	13.7% (323)	81.3% (39)	< 0.001	34.3 (16.3–72.6)	< 0.001	20.4 (7.1–58.9)
Sleep disruption	9.7% (229)	29.2% (14)	< 0.001	4.0 (2.1–7.6)		
Enlarged lymph nodes in the neck	8.1% (190)	77.1% (37)	< 0.001	47.8 (23.9–95.6)	< 0.001	96.7 (20.7–452.0)
Severe itching	6.4% (150)	50.0% (24)	< 0.001	18.1 (9.8–33.3)	< 0.001	45.9 (7.3–288.7)
Amenorrhea, abortion, or sterility*	6.1% (81/1320)	57.9% (11/19)	< 0.001	24.0 (9.4–61.7)		
Motor disorders	4.1% (97)	52.1% (25)	< 0.001	36.2 (19.2–68.2)	0.07	4.5 (0. 9–22.5)
Psychiatric problems	1.9% (45)	22.9% (11)	< 0.001	20.3 (9.5–43.4)		
Speech disorders	1.2% (27)	0.0% (0)	0.98			
Convulsions	0.8% (18)	10.4% (5)	< 0.001	20.7 (7.0–61.0)		
Coma	0.2% (4)	2.1% (1)	0.02	15.7 (1.59–155.9)		

This table shows gender, age and the frequency of clinical symptoms and signs in clinical suspects and in HAT patients. The association to HAT positivity was first assessed in univariable analysis and after using multi-variable mixed logistic regression. HAT: Human African trypanosomiasis; *OR*: Odds ratio; *CI*: Confidence interval; * only females considered, the numbers used to estimate the frequency are shown in the brackets

### HAT status of study participants

For further analysis of the results, study participants were considered as true HAT positives if they were confirmed as HAT patients based on trypanosome observation during microscopy performed on blood, lymph, or CSF specimens (*n* = 48). Clinical suspects were considered as true HAT negatives (*n* = 2297) if they either (i) tested negative to all 3 RDTs (*n* = 2231); or (ii) were RDT positive, but parasite negative in microscopy (*n* = 66). The latter group included subjects (Fig. 1) who had all 4 reference laboratory tests negative (*n* = 37); subjects that did not undergo reference laboratory tests (*n* = 23); subjects that were reference laboratory test positive but in whom, upon re-examination with microscopy, no parasites could be found (*n* = 4); and subjects that were reference laboratory test positive but died before a second parasitological tests could be carried out (*n* = 2). The 8 RDT positives who were completely lost to follow-up and did not undergo any parasitology were excluded from further analyses.

### Clinical symptoms and signs associated with HAT, regression analysis

**Fig. 2:**
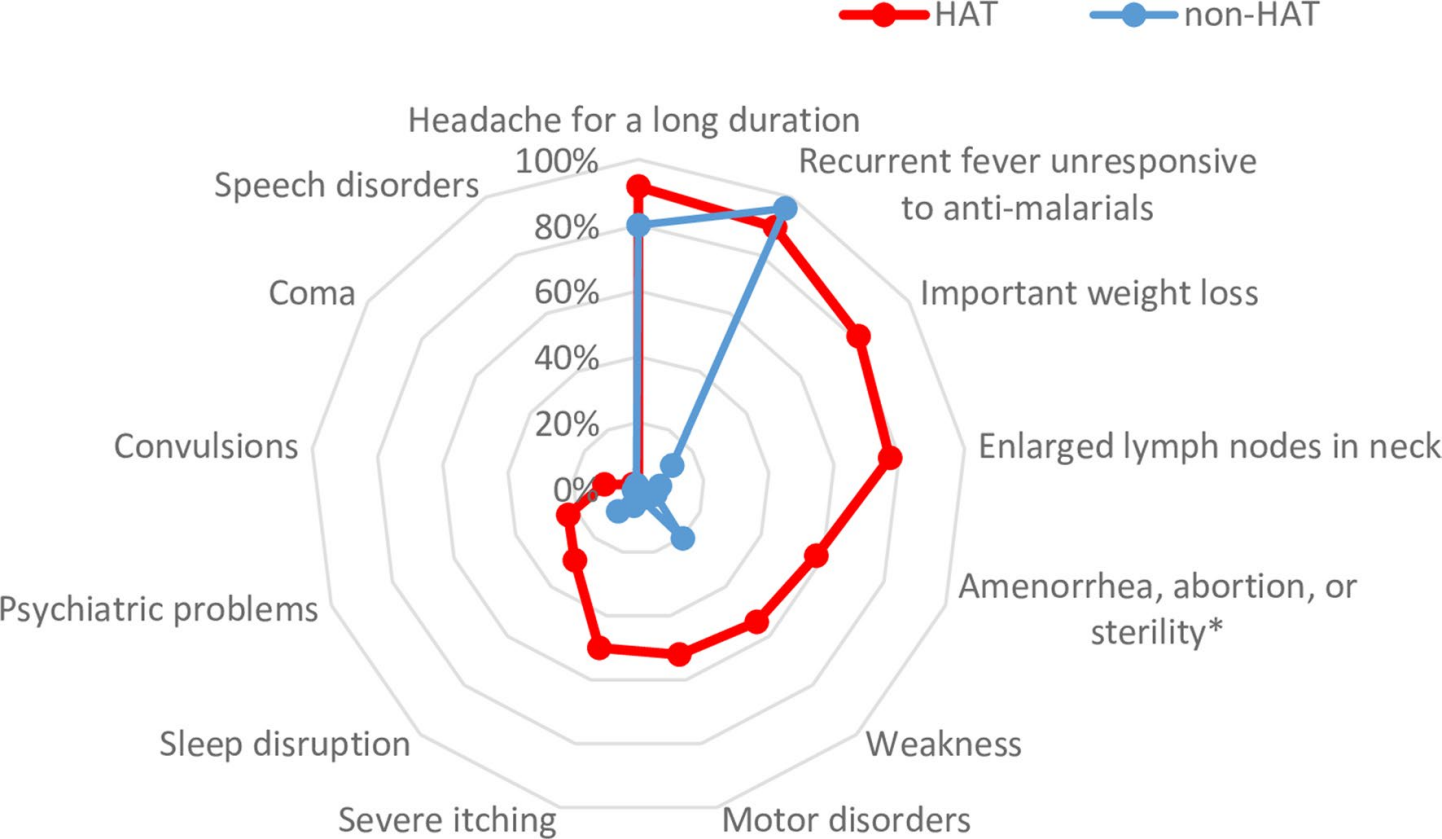
Frequency of 13 clinical symptoms and signs in human African trypanosomiasis (HAT) and non-HAT affected study participants. The figure contains data for 48 HAT and 2297 non-HAT participants with the exception of * only 19 HAT and 1294 non-HAT female participants

The frequency of the different inclusion clinical symptoms and signs, in HAT (*n* = 48) and non-HAT affected study participants (*n* = 2297), is summarized in Fig. 2.

Convulsions were highly correlated with coma (Spearman's rho ρ = 0.87) and motor disorders (ρ = 0.63). Coma was also correlated with motor disorders (ρ = 0.70) and enlarged lymph nodes in the neck (ρ = 0.61). The results of the unconditional associations between the explanatory variables gender, age and clinical parameters, and the dependent variable HAT positivity, are presented in Table 1. While amenorrhea, abortion, or sterility was univariably associated with HAT positivity, it was not included in the multivariable model since it only related to female participants. The final multivariable logistic regression model for HAT patients included five explanatory variables (gender, enlarged lymph nodes in the neck, important weight loss, severe itching, and motor disorders) and two significant interaction terms (enlarged lymph nodes in the neck × severe itching and important weight loss × motor disorders). Clinical suspects presenting with enlarged lymph nodes in the neck had the highest odds (96.74) to have HAT, followed by those that presented with severe itching or important weight loss. Males had a higher odd of having HAT compared to females. Odds of clinical suspects presenting with both enlarged lymph nodes in the neck and severe itching (compared to a participant who had neither enlarged lymph nodes in the neck nor severe itching) increased to 413 (*P* = 0.03). Similarly, when both important weight loss and motor disorders were present (compared to a participant who had neither), the odds of being HAT positive increased to 2220 (*P* = 0.01). The intra-cluster correlation coefficient was 5.26 × 10^–12^, suggesting that spatial clustering within prefectures was negligible.

### Diagnostic performance of clinical presentation

The diagnostic performance of (co-)occurrence of the 4 clinical symptoms and signs that were associated singly or in combination with HAT, namely enlarged lymph nodes in the neck, severe itching, important weight loss and motor disorders, was studied in function of the HAT status in 48 HAT and 2297 non-HAT affected study participants (Table 2). Presence of enlarged lymph nodes in the neck, and/or important weight loss and/or severe itching and/or motor disorders had 97.9% sensitivity (only 1/42 HAT patients did not have any of these 4 symptoms or signs) and 75.6% specificity. Although the PPV of observing at least one symptom or sign remained limited to 7.7%, this increased to 39.3% when co-existence of 2 or more of the 4 selected clinical presentations were considered.

**Table 2:** The diagnostic performance of occurrence of 4 key clinical presentations for human African trypanosomiasis (HAT) diagnosis

Number of signs or symptoms present	% Sensitivity (*n*/*N*, 95% *CI*)	% Specificity (*n*/*N*, 95% *CI*)	% PPV (*n*/*N*, 95% *CI*)	% NPV (*n*/*N*, 95% *CI*)	% Accuracy (*n*/*N*, 95%* CI*)
≥ 1/4	97.9 (47/48, 88.9–99.9)	75.6 (1737/2297, 73.8–77.4)	7.7 (47/607, 5.7–10.2)	99.9 (1737/1738, 99.7–100)	76.1 (1784/2345, 74.3–77.8)
≥ 2/4	87.5 (42/48, 74.8–95.3)	97.2 (2232/2297, 96.4–97.8)	39.3 (42/107, 30.1–49.2)	99.7 (2232/2238, 99.4–99.9)	97.0 (2274/2345, 96.2–97.6)
≥ 3/4	56.3 (27/48, 41.2–70.5)	99.7 (2289/2297, 99.3–99.9)	77.1 (27/35, 59.9–89.6)	99.1 (2298/2310, 98.6–99.4)	98.8 (2316/2345, 98.2–99.2)
4/4	18.8 (9/48, 9.0–32.6)	100 (2297/2297, 99.8–100)	100 (9/9, 66.4–100)	98.3 (2297/2336, 97.7–98.8)	98.3 (2306/2345, 97.7–98.8)

Sensitivity, specificity, positive predictive value (PPV) and negative predictive value (NPV) of occurrence of presence of enlarged lymph nodes in the neck and/or important weight loss and/or severe itching, and/or motor disorders were determined for identification of HAT patients. The occurrence of at least one symptom or sign (≥ 1/4) and co-occurrence (≥ 2/4; ≥ 3/4 or all 4/4) of the 4 selected clinical presentations was counted in 48 HAT patients and 2297 non-HAT affected study participants, and proportions (*n*/*N*) with 95% confidence intervals (*CI*) were determined

### Diagnostic performance of rapid diagnostic tests

**Fig. 3:**
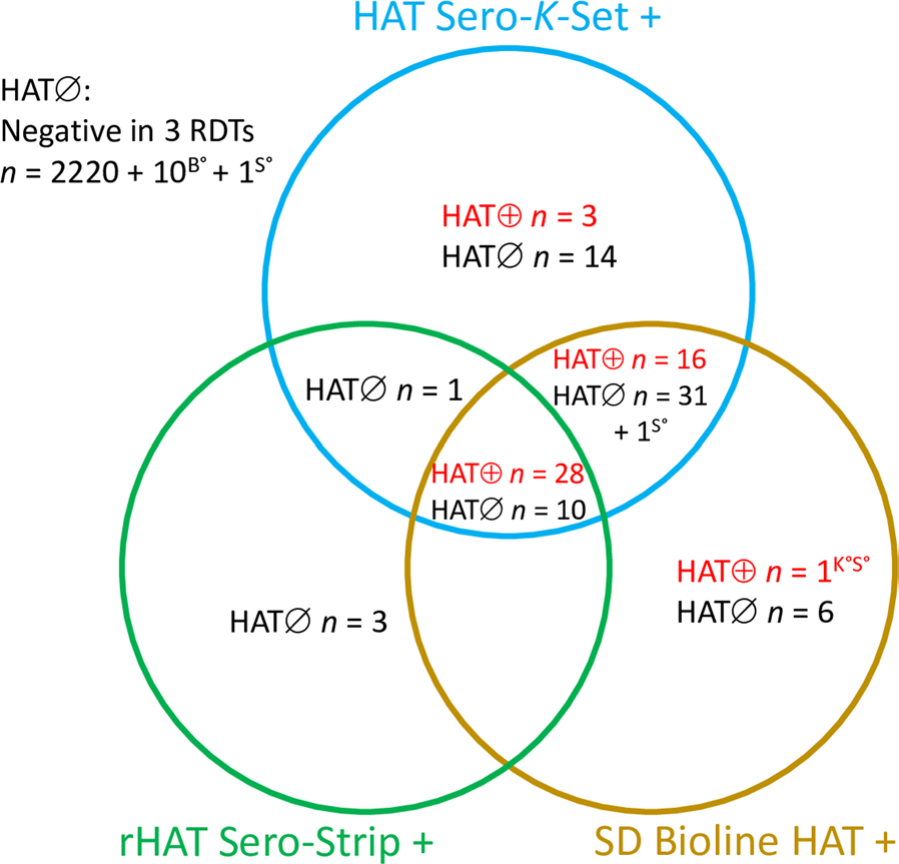
Individual RDT results of human African trypanosomiasis (HAT) patients and HAT free participants. The Venn diagram shows results in the RDTs HAT Sero-K-Set, rHAT Sero-Strip and SD Bioline HAT of 48 HAT patients and 2297 HAT free participants. ^K°^ HAT Sero-*K*-Set not performed, ^S°^ rHAT Sero-Strip not performed, ^B°^ SD Bioline HAT not performed, HAT ⊕ : HAT patients, HAT∅: HAT free participants

For estimation of the RDT diagnostic performance in 48 HAT and 2297 non-HAT clinical suspects, a few participants had partially missing RDT results (Table 3, Fig. 3). Of the three RDTs, HAT Sero-*K*-Set had the highest sensitivity (100%), followed by SD Bioline HAT (93.8%) and rHAT Sero-Strip (59.6%). The highest specificity was observed with rHAT Sero-Strip (99.4%), while HAT Sero-*K*-Set and SD Bioline HAT had similar specificities of 97.5% and 97.9% respectively. The PPV of the individual RDTs ranged between 45.2% and 66.7%, while the NPV was between 99.2% and 100%. Using the RDTs in parallel resulted in a high sensitivity (93.8–100%), specificity (97.1–97.7%) and NPV (99.9–100%), while the PPV was limited (42.1–46.4%). In series combinations including rHAT Sero-Strip led to low sensitivities (59.6%), except for the HAT Sero-*K*-Set and SD Bioline HAT combination (93.6%). There was moderate agreement between HAT Sero-*K*-Set and rHAT Sero-Strip (Kappa = 0.52; 0.43–0.62; SE = 0.02), and rHAT Sero-Strip and SD Bioline HAT (Kappa = 0.55; 0.45–0.65; SE = 0.02), while the agreement between HAT Sero-*K*-Set and SD Bioline HAT was almost perfect (Kappa = 0.86; 0.81–0.92; SE = 0.02).

**Table 3:** The diagnostic performance of 3 rapid diagnostic tests for diagnosis of human African trypanosomiasis (HAT)

	% Sensitivity (*n*/*N*, 95% *CI*)	% Specificity (*n*/*N*, 95% *CI*)	% PPV (*n*/*N*, 95% *CI*)	% NPV (*n*/*N*, 95% *CI*)	% Accuracy (*n*/*N*, 95% *CI*)
HAT Sero-*K*-Set	100 (47/47, 92.5–100)	97.5 (2240/2297, 96.8–98.1)	45.2 (47/104, 35.4–55.3)	100 (2240/2240, 99.8–100)	97.6 (2287/2344, 96.9–98.2)
rHAT Sero-Strip	59.6 (28/47, 44.3–73.3)	99.4 (2281/2295, 99.0–99.7)	66.7 (28/42, 50.5–80.4)	99.2 (2281/2300, 98.7–99.5)	98.6 (2309/2342, 98.0–99.0)
SD Bioline HAT	93.8 (45/48, 82.8–98.7)	97.9 (2239/2287, 97.2–98.4)	48.4 (45/93, 37.9–59.0)	99.9 (2239/2242, 99.6–100)	97.8 (2284/2335, 97.1–98.4)

The individual diagnostic sensitivity, specificity, positive predictive value (PPV) and negative predictive value (NPV). Reference standard: Microscopic observation of trypanosomes in any body fluid. *n/N*: proportion; *CI:* Confidence interval

### Diagnostic performance of reference laboratory tests on dried blood spots

**Fig. 4:**
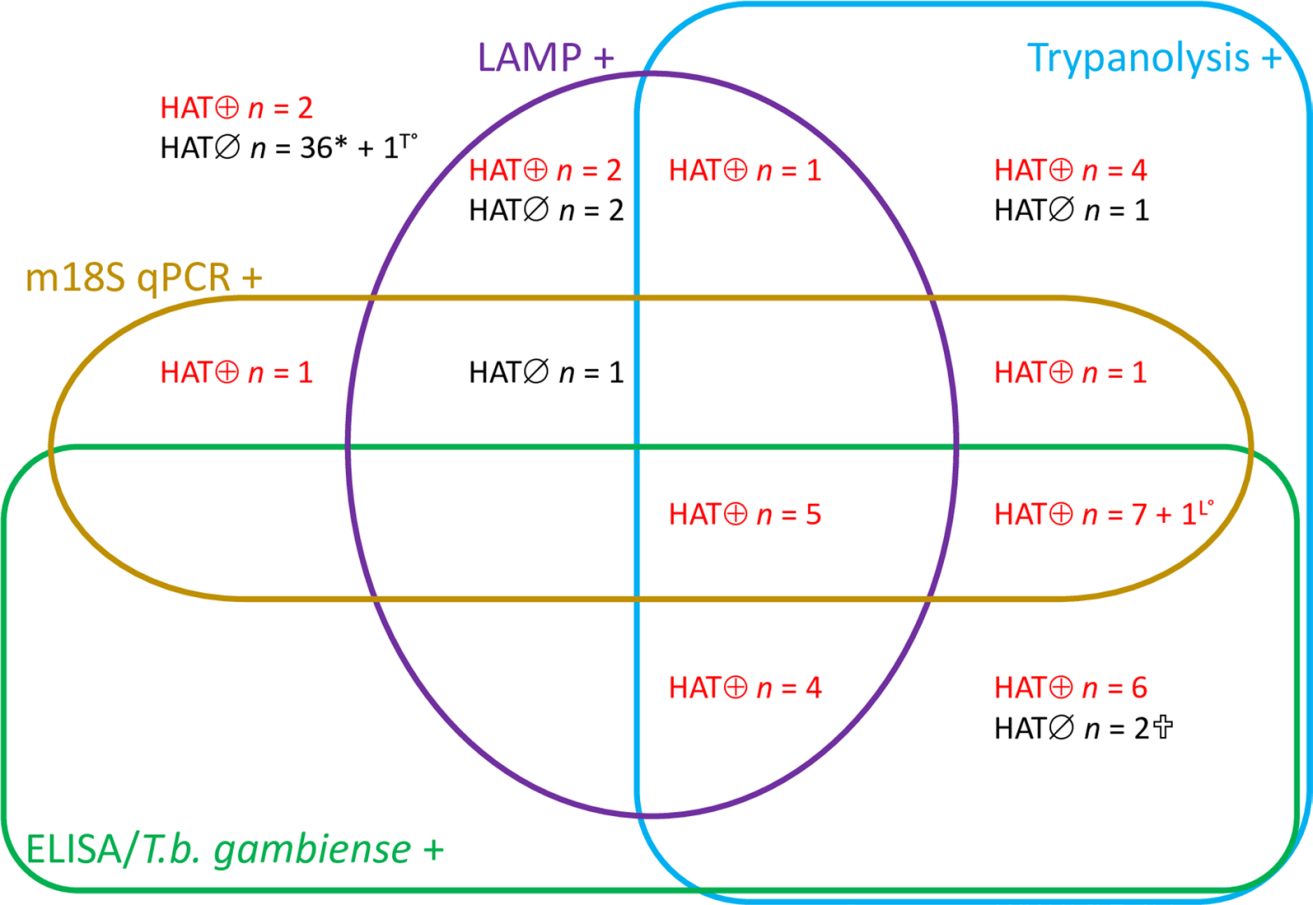
Reference laboratory test results on dried blood spots. The Venn diagram shows the results of trypanolysis, ELISA/*T.b. gambiense*, LAMP and m18S qPCR on DBS from 34 HAT patients (HAT ⊕) and 43 HAT negatives (HAT∅) who all tested rapid diagnostic test positive. ^T°^trypanolysis not performed, ^L°^LAMP not performed, ✞ died after the first parasitological examination at inclusion, *including 1 HAT negative person with 265 WBC/µl. *WBC* White blood cell

Among the 48 HAT patients, 34/48 had a DBS and all 4 DBS test results were available for 33/34, while 1/34 HAT patient missed a LAMP result. Among the 66 RDT positive HAT negatives (Fig. 1), 43/66 had DBS and all 4 DBS test results were available for 42/43, while 1/43 missed a trypanolysis result. The individual results of all DBS are shown in Fig. 4.

Table 4 summarizes the diagnostic performance of each individual reference laboratory test. Trypanolysis (in parallel on *Trypanosoma brucei gambiense* variable antigen type LiTat 1.3 and LiTat 1.5) had the highest sensitivity (85.3%), followed by ELISA/*T.b. gambiense* (67.6%). Sensitivities for m18S qPCR and LAMP were low. The highest specificity was observed for m18S qPCR (97.7%), followed by ELISA/*T.b. gambiense* (95.3%). The PPV ranged between 80.0% for LAMP and 93.8% for m18S qPCR, while the NPV ranged between 65.6% for LAMP and 88.6% for trypanolysis. There was fair agreement between LAMP and the three other reference laboratory tests [with trypanolysis: Kappa = 0.23 (0.02––0.4); SE = 0.10); with m18S qPCR: Kappa = 0.25 (0.00–0.51; SE = 0.11); with ELISA: Kappa = 0.29 (0.06–0.52; SE = 0.11)]. There was moderate agreement between m18S qPCR and both trypanolysis (Kappa = 0.42; 0.23–0.61; SE = 0.10) and ELISA/*T.b.gambiense* (Kappa = 0.51; 0.30–0.72; SE = 0.11). The agreement between ELISA/*T.b. gambiense* and trypanolysis was almost perfect (Kappa = 0.81; 0.67–0.94; SE = 0.11).

**Table 4:** The diagnostic performance of reference laboratory tests on dried blood spot for diagnosis of human African trypanosomiasis

Reference laboratory test	% Sensitivity (*n*/*N*, 95% *CI*)	% Specificity (*n*/*N*, 95% *CI*)	*% PPV (n/N,* 95%* CI)*	*% NPV (n/N, *95% *CI)*	% Accuracy (*n*/*N*, 95% *CI*)
Trypanolysis (in parallel)	85.3 (29/34, 68.9–95.0)	92.9 (39/42*, 80.5–98.5)	90.6 (29/32, 75.0–98.0)	88.6 (39/44, 75.4–96.2)	89.5 (68/76, 80.3–95.3)
TL LiTat 1.3	79.4 (27/34, 62.1–91.3)	92.9 (39/42*, 80.5–98.5)	90.0 (27/30, 73.5–97.9)	84.8 (39/46, 71.1–93.7)	86.8 (66/76, 77.1–93.5)
TL LiTat 1.5	55.9 (19/34, 37.9–72.8)	92.9 (39/42*, 80.5–98.5)	86.4 (19/22, 65.1–97.1)	72.2 (39/54, 58.4–83.8)	76.3 (58/76, 65.2–85.3)
ELISA/*T.b. gambiense*	67.6 (23/34, 49.5–82.6)	95.3 (41/43, 84.2–99.4)	92.0 (23/25, 74.0–99.0)	78.8 (41/52, 65.3–88.9)	83.1 (64/77, 72.9–90.7)
m18S qPCR	44.1 (15/34, 27.2–62.1)	97.7 (42/43, 87.7–99.9)	93.8 (15/16, 69.8–99.8)	68.9 (42/61, 55.7–80.1)	74.0 (57/77, 62.8–83.4)
LAMP	36.4 (12/33*, 20.4–54.9)	93.0 (40/43, 80.9–98.5)	80.0 (12/15, 51.9–95.7)	65.6 (40/61, 52.3–77.3)	68.4 (52/76, 56.7–78.6)

The individual diagnostic sensitivity, specificity, positive predictive value (PPV) and negative predictive value (NPV) of trypanolysis (TL), ELISA/*T.b. gambiense*, m18S qPCR and Loopamp *Trypanosoma brucei* Detection kit (LAMP) for diagnosis of HAT was determined on 77 dried blood spots collected from rapid diagnostic test positives. Reference standard: Microscopic observation of trypanosomes in any body fluid.* CI*: Cconfidence interval. * 1 DBS missing

The TgsGp-qPCR was carried out on 19 DBS only, 13 from HAT patients and 6 from RDT positive HAT negatives. Among the tested HAT patients, TgsGp-qPCR sensitivity was 38.5% (5/13, 95% *CI*: 17.7–64.5%). The TgsGp-qPCR specificity was 100% (6/6, 95% *CI*: 61.0–100.0%).

## Discussion

The HAT prevalence observed among the study participants during the 3 years of passive screening was 2.0%. The overall HAT prevalence reported by the national program in passive screening in the same prefectures in 2017 and 2018 was respectively 0.98 and 0.39% [3]. The DiTECT-HAT-WP2 study was set up in a small selection of experienced reference hospitals and health posts, including the 3 HAT reference centers that might attract relatively more HAT patients, which probably explains the difference. The relatively high prevalence allowed to successfully assess the sensitivity, specificity, positive and negative predictive value of clinical symptoms and signs, HAT rapid diagnostic tests and reference laboratory tests for diagnosis of HAT.

Although there may be geographical and stage specific variations, the clinical picture of HAT has been described in detail [9, 10]. However, within a context of passive screening for HAT, it is important to consider the frequency of signs and symptoms in non-HAT affected individuals visiting the health infrastructure as well, for proposing criteria for HAT referral. This was well illustrated in the actual study by the criterion “presence of recurrent fever not responding to anti-malarial medication”. Fever is considered among the leading symptoms of HAT [26, 27] and has previously been reported at 97% and 73.4% frequency in Guinean HAT patients [28, 29]. Recurrent fever not responding to anti-malarial medication was also one of the most frequent symptoms (89.6%) in our HAT patients but in univariable analysis, it was negatively associated with HAT and the odds that participants with fever would have HAT were below one, suggesting recurrent fever not responding to anti-malarial medication would be protective. This is probably an artefact given that it was common also in non-HAT affected participants (96.1%). Indeed, in the multivariable analysis, both recurrent fever not responding to anti-malarial medication and having a headache for a long duration (the 2 clinical signs most frequently observed in the study participants) were not statistically significant. Among the 13 clinical symptoms and signs considered as inclusion criteria, using multivariable logistic regression, we were able to identify 4 key clinical presentations which could be used to select, among a health care seeking population in HAT endemic areas in Guinea, individuals at increased risk for HAT and which should be referred for further HAT screening. The presence of either enlarged lymph nodes in the neck, and/or severe itching, and/or important weight loss and/or motor disorders was in our study 97.9% sensitive for HAT and had 75.6% specificity, resulting in a PPV of 7.7%. A combination of at least 2 signs or symptoms increased the PPV to 39.3%, but resulted in a decrease in sensitivity. In particular presence of enlarged lymph nodes in the neck has previously been identified in 90% and 93% of HAT patients in Guinea, while occurrence of itching has been reported with frequencies of 93% and 29.4% [28, 29]. In the present study, enlarged lymph nodes in the neck and severe itching were present in respectively 77.1% and 50.0% of HAT patients, and it should be underlined that severe itching was retained as an inclusion criterion in the study at the specific request of the Guinean national HAT program. Although there might be geographical variation in the clinical presentation of HAT in different HAT endemic foci, previous independent studies on clinical presentation-based HAT diagnostic referral [11] identified sleep problems, neurological problems and weight loss as core symptoms in South Sudan, with or without oedema, swollen lymph nodes or proximity to livestock. Their diagnostic algorithms, based on these clinical presentations, had sensitivities up to 92.6% and NPVs and PPVs of maximum 8.7% [11]. Although itching was also significantly associated with HAT in South Sudan, it was not retained in the algorithms. In the Republic of Congo, enlarged lymph nodes, oedema, fever, headaches and itching were considered for establishing a clinical presentation based diagnostic algorithm for identifying HAT [30]. In Côte d’Ivoire, odds for having a positive RDT for HAT were increased in study participants with sleep disturbances, motor disorders, convulsions, important weight loss and psychiatric problems. In the Ivorian part of the DiTECT-HAT-WP2 study, only 2 HAT patients were identified, and the overall frequency of enlarged lymph nodes in the neck and severe itching was low (3.6 and 8.2% respectively). Finally, our finding in the present study that males were at higher odds to have HAT than females, is in line with previous observations in Guinea, and has been linked to activities like rice growing, salt extraction, fishing and wood collection, which expose men more to the vector [29]. The hypothesis of unequal access to the health system [29], disfavouring women, can probably be excluded, as slightly more women were included in the present study. On the other hand, men traditionally participate less in active screening [29], which could explain why, once they start developing second stage HAT symptoms, they are more easily picked up through passive screening.

The combined seroprevalence during this study was 5.2%, ranging from 1.9 to 4.7% for the individual RDTs. As for the HAT prevalence, this was again higher than the overall seroprevalence of 1.72% and 0.98% previously reported in 2017 and 2018 [3]. The specificities of the 3 RDTs observed in Guinea confirm those reported in Côte d’Ivoire [21], and in other prospective evaluations in Central Africa [12, 13, 31, 32]. Despite the higher HAT prevalence in the actual study, the PPV of the 3 RDTs, ranging between 45.2% and 66.7% are similar to those observed in passive case detection in Guinea for 2017–2018 [3]. Sensitivity of HAT Sero-*K*-Set was 100%, confirming the high sensitivity for this test observed in prospective studies in the Democratic Republic of the Congo [12, 32]. For SD Bioline HAT, a wide variation of sensitivities has been reported from different prospective studies in Central Africa, ranging from 59.0% [31] over 89.3% [13] to 92.0% [33]. In a retrospective study on stored plasma originating mainly from Guinean HAT patients, a sensitivity of 99.6% was observed [19], although this might have been an overestimation due to selection bias using CATT/*T.b. gambiense*. In the present study, the 93.8% sensitivity of SD Bioline HAT was close to the higher sensitivity values reported for the Democratic Republic of the Congo [33]. The sensitivity of 59.6% observed with rHAT Sero-Strip in the present study was low compared to the > 97.5% sensitivities obtained using stored specimens in a laboratory evaluation [34]. This was the first evaluation of the sensitivity of the rHAT Sero-Strip dipstick test under field conditions and it cannot be excluded that transport stress, the higher environmental temperatures or the humidity might have affected test stability. In parallel or in series combination of tests, with or without rHAT Sero-Strip did not improve diagnostic performance, probably because of the agreement between the HAT Sero-*K*-Set and SD Bioline HAT test results.

Evaluation of the diagnostic performance of the parasitological tests was not an objective of our study and not all tests were systematically performed on all RDT positives, but our results confirm the relatively high sensitivity of lymph examination in Guinea [8, 29]. Indeed, 26/48 (54.2%) of the HAT patients had trypanosomes upon microscopic examination of the lymph node exudate.

Unfortunately, DBS were missing for a relatively high number of RDT positives. The specificity of the 4 reference laboratory tests in this study was similar as for passive screening in Côte d’Ivoire [21]. For ELISA/*T.b. gambiense* and trypanolysis, specificity was lower than in active screening in Burkina Faso [35]. However, among the 6 DBS positives considered as non-HAT for determination of the diagnostic performance (Figs. 1 and 4), two participants died before they could be re-examined. As these participants had one negative microscopic examination, we could not determine the exact cause of death and as we cannot exclude that the positive test results were due to cross reactivity, we preferred not to exclude these subjects from the study. They had respectively 2/4 and 3/4 key symptoms and signs, were positive in 3/3 and 2/3 RDTs and were both positive in trypanolysis and ELISA/*T.b. gambiense* but not in the molecular tests. It cannot be confirmed nor excluded that these were real HAT patients, thus the specificity values of trypanolysis and ELISA/*T.b. gambiense* might have been underestimated. The sensitivities of trypanolysis and ELISA/*T.b. gambiense* observed in the present study were modest. It has previously been demonstrated that the sensitivity of trypanolysis and inhibition ELISA is lower on DBS compared to plasma [14, 36]. In DR Congo, trypanolysis on DBS was 95.1% sensitive [37], while ELISA/*T.b. gambiense* was estimated to be 82.2% sensitive [38]. The low sensitivities for the molecular tests LAMP and m18S qPCR were not a complete surprise. Firstly, DBS have been shown to be suboptimal for PCR and better results are obtained with nucleic acid preservation in different types of stabilisation buffers [37, 39]. Secondly, LAMP and m18S qPCR on DBS have been shown to have limited analytical sensitivity (100 and 1000 trypanosomes/ml respectively) [18], which is lower than that of mAECT-BC (10 trypanosomes/ml) [8], which was used together with lymph and CSF examination, to diagnose HAT in the present study. Finally, a prolonged or suboptimal storage of DBS could also have affected the sensitivity of both the serological and the molecular reference laboratory tests: DBS were not systematically shipped to the reference laboratory (median delay of 3 months, with a maximum delay of up to 9 months), and exposure to humidity, despite storage with silica gel, cannot be entirely excluded.

Some limitations of this multi-country study have already been discussed in detail elsewhere [21], including non-inclusion of individuals without symptoms, incomplete inclusion of individuals presenting with symptoms or signs at the CDT or SSS, the assumption that individuals testing negative in all 3 RDTs are not affected by HAT without carrying out parasitological examinations, and imperfect sensitivity of parasitological techniques used as a gold standard. A number of additional limitations apply to the present study in Guinea. All the 48 HAT patients that were diagnosed suffered from stage 2 HAT. This is a known problem inherent to passive screening [3], not only in Guinea [11, 26, 27, 40]. As a result, the proposed key clinical presentations might have high sensitivity for stage 2 HAT, but their real ability to pick up stage 1 HAT patients, which may be asymptomatic or have only mild symptoms, remains to be determined. No stage 1 patients have been diagnosed in Guinea in the last 3 years. This could also be a consequence of the vector control program that has been rolled out in all endemic districts since 2017, impacting disease transmission. Furthermore, severe itching and enlarged lymph nodes in the neck in particular are relatively well known as clinical symptoms and signs of HAT in Guinea. It is possible that clinicians responsible for including participants gave more attention to these clinical presentations, compared to others which could have remained under-detected. The clinical presentation was considered as a rapid and simple decision tool for inclusion and referral to more specific HAT RDTs, and no attempts were made to quantify signs or symtoms. Some presentations, like psychiatric problems, motor disorders and speech disorders were grouped. Being relatively imprecise, we can therefore not exclude that the clinical data have been influenced by a level of subjectivity by the patient or the clinician, or by the clinician’s skills. Moreover, inclusion of participants in the study was not done by expert clinicians but by local health workers from the health centres. However, in particular for important weight loss, severe itching and motor disorders, detailed clinical data collected in therapeutic trials carried out on Guinean HAT patients could be consulted retrospectively [41]. Many DBS of RDT positives were missing, leading to relatively large confidence intervals in diagnostic test performance estimations. Health workers from the selected health centres collected the DBS and they had a lot of work in addition to the study. They may have been overloaded and forgot to take DBS. In addition, at the beginning of the study, sometimes an insufficient volume of blood was spotted and these DBS had to be discarded. Moreover, the delay between collection and analysis of DBS might have affected sensitivity of the tests. Finally, newer second generation RDTs were not available during the study, in particular SD Bioline HAT 2.0 with recombinant antigens, despite its large scale evaluation in the Democratic Republic of the Congo prior to the start of the present study [31]. Production of rHAT Sero-Strip has since been discontinued, while SD Bioline HAT with native antigens is nowadays unavailable.

The actual study also has important strengths. The high HAT prevalence in Guinea allowed us to assess the association between clinical symptoms and signs and HAT in Guinea and the diagnostic performance of the combination of 4 key clinical presentations, 3 RDTs, and consequent reference laboratory tests on DBS of RDT positives. A similar study in Côte d’Ivoire [21] allowed us to associate the clinical picture with RDT positivity and to assess test specificity of RDTs and reference laboratory tests, but included only 2 HAT patients. The Guinean HAT control program paid particular attention to actively retrieving RDT positives, resulting in a limited loss to follow-up.

## Conclusions

In passive case detection, we can propose to health workers and clinicians in Guinean HAT endemic areas a relatively simple set of criteria with high sensitivity for selecting individuals to be further tested using HAT RDTs, which would result in a reduction of almost 70% of the HAT RDTs to be carried out. Performance of both HAT Sero-*K*-Set and SD Bioline HAT is sufficient for referring RDT positives for microscopic examination. Taking into account the future availability of non-toxic oral drugs to treat both stages of HAT [41], a target product profile has been established by the World Health Organization for a gambiense HAT test to identify individuals with suspected but microscopically unconfirmed g-HAT infection, eligible for treatment [42]. In HAT endemic areas in Guinea, both RDTs largely meet the minimum requirements for specificity, while HAT Sero-*K*-Set meets the desirable and SD Bioline HAT approaches the minimal sensitivity. Testing of DBS may discriminate individuals with false positive RDTs from true HAT patients which need to be re-examined for confirmation of HAT, and could become important to know the epidemiological HAT status of specific areas in the future. All DBS tests showed sufficient specificity, but only the antibody detection tests, and in particular trypanolysis, had sufficient sensitivity. More care should however be given to correct collection, storage, and shipment of DBS, and to minimising the delay between collection and testing of the specimens.

In the future, priority should be given to assessing the diagnostic performance of new generation RDTs and of new reference laboratory tests. Inhibition ELISA could replace trypanolysis [36], increasing the feasibility of implementation of DBS testing in-country and reducing DBS storage time. New nucleic acid detection tests including SHERLOCK [43] or (RT)-qPCRs [39] should be evaluated prospectively. Decisions for the most suitable diagnostic algorithm for passive HAT case detection in HAT endemic areas in Guinea should also be guided by cost-effectiveness analysis.

## Data Availability

The public sharing of personal health data is subject to the General Data Protection Regulation. The health data underlying the findings described in the manuscript can therefore not be made public. Metadata are available via Lejon V, Camara O, Camara M, Ilboudo H, Kaboré J, Compaoré CFA, Buscher P, Bucheton B, 2022, "Passive case detection of Human African Trypanosomiasis in Guinea: symptoms and signs, rapid diagnostic test results and laboratory test results", DataSuds, https://doi.org/10.23708/ZDD0OW [44]. The datasets generated and analysed in the present manuscript will be made available to qualified researchers upon request and after signing a confidentiality agreement. Data requests may be sent to the Institut de Recherche pour le Développement (IRD) data administrator (data@ird.fr).

## Abbreviations

CDT: Centre for diagnosis and treatment
CSF: Cerebrospinal fluid
DBS: Dried blood spot
DiTECT-HAT-WP2: Diagnostic tools for human African trypanosomiasis elimination and clinical trials work package 2, passive case detection
ELISA: Enzyme-linked immunoassay
HAT: Human African trypanosomiasis
LAMP: Loopamp *Trypanosoma brucei* Detection kit
mAECT-BC: Mini anion exchange centrifugation technique on buffy coat
NPV: Negative predictive value
PPV: Positive predictive value
RDT: Rapid diagnostic test
SSS: Serological screening sites
qPCR: Quantitative polymerase chain reaction

## References

[1] Franco JR, Cecchi G, Paone M, Diarra A, Grout L, Kadima Ebeja A (2022). The elimination of human African trypanosomiasis: achievements in relation to WHO road map targets for 2020. PLoS Negl Trop Dis.

[2] Kagbadouno MS, Camara M, Rouamba J, Rayaisse JB, Traoré IS, Camara O (2012). Epidemiology of sleeping sickness in Boffa (Guinea): where are the trypanosomes?. PLoS Negl Trop Dis.

[3] Camara O, Biéler S, Bucheton B, Kagbadouno M, Mathu Ndung’u J, Solano P (2021). Accelerating elimination of sleeping sickness from the Guinean littoral through enhanced screening in the post-Ebola context: a retrospective analysis. PLoS Negl Trop Dis.

[4] Kagbadouno M, Camara O, Camara M, Ilboudo H, Camara M, Rayaisse JB (2018). Ebola outbreak brings to light an unforeseen impact of tsetse control on sleeping sickness transmission in Guinea. BioRxiv.

[5] Snijders R, Fukinsia A, Claeys Y, Hasker E, Mpanya A, Miaka E (2021). Costs and outcomes of integrated human African trypanosomiasis surveillance system using rapid diagnostic tests, Democratic Republic of the Congo. Emerg Infect Dis.

[6] Camara M, Ouattara E, Duvignaud A, Migliani R, Camara O, Leno M (2017). Impact of the Ebola outbreak on *Trypanosoma brucei gambiense* infection medical activities in coastal Guinea, 2014–2015: a retrospective analysis from the Guinean national Human African Trypanosomiasis control program. PLoS Negl Trop Dis.

[7] World Health Organization. Control of Neglected Tropical Diseases. NTDs & COVID-19: https://www.who.int/teams/control-of-neglected-tropical-diseases/overview/ntds-and-covid-19. Accessed 21 Nov 2021.

[8] Camara M, Camara O, Ilboudo H, Sakande H, Kaboré J, N’Dri L (2010). Sleeping sickness diagnosis: use of buffy coats improves the sensitivity of the mini anion exchange centrifugation test. Trop Med Int Health.

[9] Boa YF, Traore MA, Doua F, Kouassi-Traore MT, Kouassi BE, Giordano C (1988). The different present-day clinical picture of human African trypanosomiasis caused by *T. b. gambiense*. Analysis of 300 cases from a focus in Daloa, Ivory Coast. Bull Soc Pathol Exot Filiales..

[10] Blum J, Schmid C, Burri C (2006). Clinical aspects of 2541 patients with second stage human African trypanosomiasis. Acta Trop.

[11] Palmer JJ, Surur EI, Goch GW, Mayen MA, Lindner AK, Pittet A (2013). Syndromic algorithms for detection of gambiense human African trypanosomiasis in South Sudan. PLoS Negl Trop Dis.

[12] Büscher P, Mertens P, Leclipteux T, Gilleman Q, Jacquet D, Mumba-Ngoyi D (2014). Sensitivity and specificity of HAT Sero-*K*-SeT, a rapid diagnostic test for serodiagnosis of sleeping sickness caused by *Trypanosoma brucei gambiense*: a case-control study. Lancet Glob Health.

[13] Bisser S, Lumbala C, Nguertoum E, Kande V, Flevaud L, Vatunga G (2016). Sensitivity and specificity of a prototype rapid diagnostic test for the detection of *Trypanosoma brucei gambiense* infection: a multi-centric prospective study. PLoS Negl Trop Dis.

[14] Camara O, Camara M, Lejon V, Ilboudo H, Sakande H, Léno M (2014). Immune trypanolysis test with blood spotted on filter paper for epidemiological surveillance of sleeping sickness. Trop Med Int Health.

[15] Mumba D, Bohorquez E, Messina J, Kande V, Taylor SM, Tshefu AK (2011). Prevalence of human African trypanosomiasis in the Democratic Republic of the Congo. PLoS Negl Trop Dis.

[16] Inocencio da Luz R, Phanzu DM, Kiabanzawoko ON, Miaka E, Verlé P, De Weggheleire A (2021). Feasibility of a dried blood spot strategy for serological screening and surveillance to monitor elimination of human African trypanosomiasis in the Democratic Republic of the Congo. PLoS Negl Trop Dis.

[17] Wamboga C, Matovu E, Bessell PR, Picado A, Biéler S, Ndung’u JM (2017). Enhanced passive screening and diagnosis for gambiense human African trypanosomiasis in north-western Uganda—moving towards elimination. PLoS ONE.

[18] Compaoré CAF, Ilboudo H, Kaboré J, Kaboré JW, Camara O, Bamba M (2020). Analytical sensitivity of loopamp and quantitative real-time PCR on dried blood spots and their potential role in monitoring human African trypanosomiasis elimination. Exp Parasitol.

[19] Jamonneau V, Camara O, Ilboudo H, Peylhard M, Koffi M, Sakande H (2015). Accuracy of individual rapid tests for serodiagnosis of gambiense sleeping sickness in West Africa. PLoS Negl Trop Dis.

[20] Büscher P, Mumba Ngoyi D, Kaboré J, Lejon V, Robays J, Jamonneau V (2009). Improved models of mini anion exchange centrifugation technique (mAECT) and modified single centrifugation (MSC) for Sleeping sickness diagnosis and staging. PLoS Negl Trop Dis.

[21] Koné M, Kaba D, Kaboré J, Thomas LF, Falzon LC, Koffi M (2021). Passive surveillance of human African trypanosomiasis in Côte d’Ivoire: understanding prevalence, clinical symptoms and signs, and diagnostic test characteristics. PLoS Negl Trop Dis.

[22] Hasker E, Kwete J, Inocencio da Luz R, Mpanya A, Bebronne N, Makabuza J (2018). Innovative digital technologies for quality assurance of diagnosis of human African trypanosomiasis. PLoS Negl Trop Dis.

[23] Dohoo I, Martin W, Stryhn H (2003). Veterinary epidemiologic research.

[24] Chen JJ (2003). Communicating complex information: The interpretation of statistical interaction in multiple logistic regression analysis. Am J Public Health.

[25] Landis JR, Koch GG (1977). The measurement of observer agreement for categorical data. Biometrics.

[26] World Health Organization. Control and surveillance of human African trypanosomiasis: report of a WHO Expert Committee. Geneva: World Health Organization; 2013 https://apps.who.int/iris/handle/10665/95732. Accessed 21 Nov 2022. 24552089

[27] Hasker E, Lumbala C, Mbo F, Mpanya A, Kande V, Lutumba P (2011). Health care-seeking behaviour and diagnostic delays for human African trypanosomiasis in the Democratic Republic of the Congo: Diagnosing HAT in DRC. Trop Med Int Health.

[28] Camara M, Kaba D, KagbaDouno M, Sanon JR, Ouendeno FF, Solano P (2005). Human African trypanosomiasis in the mangrove forest in Guinea: epidemiological and clinical features in two adjacent outbreak areas. Med Trop.

[29] Vanhecke C, Guevart E, Ezzedine K, Receveur MC, Jamonneau V, Bucheton B (2010). Human African trypanosomiasis in mangrove epidemiologic area. Presentation, diagnosis and treatment in Guinea, 2005–2007. Pathol Biol.

[30] Jannin J, Moulia-Pelat JP, Chanfreau B, Penchenier L, Louis JP, Nzaba P, et al. African human trypanosomiasis: study of a scoring system of presumptive diagnosis in the Congo. Bull World Health Organ. 1993;71:215 (In French). PMC2393445 8490985

[31] Lumbala C, Biéler S, Kayembe S, Makabuza J, Ongarello S, Ndung’u JM (2018). Prospective evaluation of a rapid diagnostic test for *Trypanosoma brucei gambiense* infection developed using recombinant antigens. PLoS Negl Trop Dis.

[32] Boelaert M, Mukendi D, Bottieau E, Kalo Lilo JR, Verdonck K, Minikulu L (2018). A phase III diagnostic accuracy study of a rapid diagnostic test for diagnosis of second-stage human African trypanosomiasis in the Democratic Republic of the Congo. EBioMedicine.

[33] Lumbala C, Bessell PR, Lutumba P, Baloji S, Biéler S, Ndung’u JM (2017). Performance of the SD BIOLINE® HAT rapid test in various diagnostic algorithms for gambiense human African trypanosomiasis in the Democratic Republic of the Congo. PLoS ONE.

[34] Büscher P, Gilleman Q, Lejon V (2013). Rapid diagnostic test for sleeping sickness. N Engl J Med.

[35] Compaoré CFA, Kaboré J, Ilboudo H, Thomas LF, Falzon LC, Bamba M (2022). Monitoring the elimination of *gambiense* human African trypanosomiasis in the historical focus of Batié, South-West Burkina Faso. Parasite.

[36] Geerts M, Van Reet N, Leyten S, Berghmans R, Rock KS, Coetzer THT (2021). *Trypanosoma brucei gambiense* -iELISA: a promising new test for the post-elimination monitoring of human African trypanosomiasis. Clin Infect Dis.

[37] Mumba Ngoyi D, Ali Ekangu R, Mumvemba Kodi MF, Pyana PP, Balharbi F, Decq M (2014). Performance of parasitological and molecular techniques for the diagnosis and surveillance of gambiense sleeping sickness. PLoS Negl Trop Dis.

[38] Hasker E, Lutumba P, Mumba D, Lejon V, Büscher P, Kande V (2010). Diagnostic accuracy and feasibility of serological tests on filter paper samples for outbreak detection of *T.b. gambiense* human African trypanosomiasis. Am J Trop Med Hyg.

[39] Ngay Lukusa I, Van Reet N, Mumba Ngoyi D, Miaka EM, Masumu J, Patient Pyana P (2021). Trypanosome SL-RNA detection in blood and cerebrospinal fluid to demonstrate active gambiense human African trypanosomiasis infection. PLoS Negl Trop Dis.

[40] Pepin J, Guern C, Milord F, Bokelo M (1989). Integration of African human trypanosomiasis control in a network of multipurpose health centers. Bull World Health Organ.

[41] Kande Betu Kumeso V, Kalonji Mutombo W, Rembry S, Valverde Mordt O, Ngolo Tete D, Prêtre A (2022). Efficacy and safety of acoziborole in patients with human African trypanosomiasis caused by *Trypanosoma brucei gambiense*: a multicentre, open-label, single-arm, phase 2/3 trial. Lancet Infect Dis.

[42] World Health Organization. Target product profile for a gambiense human African trypanosomiasis test to identify individuals to receive widened treatment. 2022: https://www.who.int/publications/i/item/9789240043299. Accessed 21 Nov 2022.

[43] Sima N, Dujeancourt-Henry A, Perlaza BL, Ungeheuer MN, Rotureau B, Glover L (2022). SHERLOCK4HAT: a CRISPR-based tool kit for diagnosis of human African trypanosomiasis. Biomedicine.

[44] Lejon V, Camara O, Camara M, Ilboudo H, Kaboré J, Compaoré CFA, et al. Passive case detection of human African trypanosomiasis in Guinea: symptoms and signs, rapid diagnostic test results and laboratory test results. DataSuds; 2022.

